# Developmental regulation of GABAergic signalling in the hippocampus of neuroligin 3 R451C knock-in mice: an animal model of Autism

**DOI:** 10.3389/fncel.2013.00085

**Published:** 2013-06-04

**Authors:** Rocco Pizzarelli, Enrico Cherubini

**Affiliations:** Department of Neuroscience, Scuola Internazionale Superiore di Studi AvanzatiTrieste, Italy

**Keywords:** neuroligin 3 mutation, developing hippocampus, GDPs, miniature GABAergic events, autism

## Abstract

Autism Spectrum Disorders (ASDs) comprise an heterogeneous group of neuro-developmental abnormalities, mainly of genetic origin, characterized by impaired social interactions, communications deficits, and stereotyped behaviors. In a small percentage of cases, ASDs have been found to be associated with single mutations in genes involved in synaptic function. One of these involves the postsynaptic cell adhesion molecule neuroligin (NL) 3. NLs interact with presynaptic neurexins (Nrxs) to ensure a correct cross talk between post and presynaptic specializations. Here, transgenic mice carrying the human R451C mutation of *Nlgn3*, were used to study GABAergic signaling in the hippocampus early in postnatal life. Whole cell recordings from CA3 pyramidal neurons in slices from NL3^R451C^ knock-in mice revealed an enhanced frequency of Giant Depolarizing Potentials (GDPs), as compared to controls. This effect was probably dependent on an increased GABAergic drive to principal cells as demonstrated by the enhanced frequency of miniature GABA_A_-mediated (GPSCs), but not AMPA-mediated postsynaptic currents (EPSCs). Changes in frequency of mGPSCs were associated with an acceleration of their decay kinetics, in the absence of any change in unitary synaptic conductance or in the number of GABA_A_ receptor channels, as assessed by peak scaled non-stationary fluctuation analysis. The enhanced GABAergic but not glutamatergic transmission early in postnatal life may change the excitatory/inhibitory balance known to play a key role in the construction and refinement of neuronal circuits during postnatal development. This may lead to behavioral deficits reminiscent of those observed in ASDs patients.

## Introduction

Autism Spectrum Disorders (ASDs) comprise a heterogeneous group of pathological conditions characterized by impaired social interactions, deficits in verbal and non verbal communication, limited interest in the surrounding environment associated with stereotyped and repetitive behavior (American Psychiatric Association, 2000). ASDs are among the most heritable neuro-developmental disorders with a high incidence in infancy and early childhood (Weintraub, 2011). It is believed that the genetic predisposition together with environmental factors contribute to alter normal brain development leading to an impaired connectivity between brain regions, ultimately weakening the specialized functions of cortical areas (Geschwind and Levitt, 2007).

Interestingly, a small percentage of cases with idiopathic ASDs have been found to be associated with single mutations in genes involved in synapses organization, pointing to synaptic dysfunction as one possible cause of autism (Südhof, 2008). Among these, mutations of genes encoding for cell adhesion molecules of the neuroligin (NL; Jamain et al., 2003; Laumonnier et al., 2004; Yan et al., 2005), neurexin (Nrx; Kim et al., 2008) families or for SHANK3, a scaffold protein involved in the structural organization of dendritic spines and a binding partner of NLs (Durand et al., 2007), have received particular attention. Nrxs and NLs bridge the cleft thus providing the functional link between pre- and post-synaptic elements of the synapse. Over-expression of NLs in non-neuronal cells co-cultured with neurons induces structural differentiation of both excitatory and inhibitory presynaptic terminals in contacting axons (Scheiffele et al., 2000; Fu et al., 2003; Graf et al., 2004; Sara et al., 2005). Conversely, Nrxs trigger postsynaptic differentiation by aggregating NLs and neurotransmitter receptors on the dendritic surface (Graf et al., 2004). The bidirectional signaling through the NL-Nrx is crucial for synapse development and stabilization (Scheiffele et al., 2000; Varoqueaux et al., 2006; Chubykin et al., 2007; Ko et al., 2009; Poulopoulus et al., 2009).

Interestingly, one missense mutation causing R451C substitution within a highly conserved region of the extracellular esterase-homology domain of the *Nlgn3* gene was detected in two male siblings, one with autism, severe intellectual disabilities and seizures and the other with Asperger syndrome (Jamain et al., 2003). To gain insights into the possible mechanisms of ASDs, this mutation was introduced into the endogenous *Nlgn3* in mice by gene targeting (Tabuchi et al., 2007). Previous work from juvenile and adult NL3^R451C^ knock-in mice have revealed deficits in social interaction, reminiscent of those found in ASDs patients, associated with modifications in GABAergic (Tabuchi et al., 2007) and glutamatergic (Etherton et al., 2011) synaptic transmission.

These studies, however, did not address the question whether the R451C mutation of NL3 affects GABAergic signaling at early developmental stages when GABA exerts a critical role in synapse formation and stabilization (Pizzarelli and Cherubini, 2011). This information is important because developmental disorders such as ASDs can be diagnosed early in infancy when an immediate therapeutic intervention may maximize potential benefits.

We examined synaptic transmission in the CA3 hippocampal area during the first 2 weeks of postnatal life when rapid morphological changes occur at both pre and postsynaptic levels (Amaral and Dent, 1981) and in adulthood. We found that the R451C mutation selectively affects GABAergic signaling and correlated network activity from birth.

## Materials and methods

### Ethical approval

All experiments were performed in accordance with the European Community Council Directive of November 24, 1986 (86/609EEC) and were approved by the local authority veterinary service and by SISSA ethical committee. All efforts were made to minimize animal suffering and to reduce the number of animal used.

### Animals

NL3^R451C^ mice (Tabuchi et al., 2007) were purchased from Jackson Laboratories (Maine, USA). Experiments were performed on off-spring male derived from heterozygous mating. Electrophysiological experiments were performed and analyzed blind before genotyping. This was carried out on tail biopsy DNA by PCR using a standard protocol. At least three mice for each genotype were used in a given experiment.

### Hippocampal slice preparation

Transverse hippocampal slices (300–400 μm tick) were obtained from neonatal (postnatal day 4–9) young (postnatal day 11–15) and adult (postnatal day 27–35) mice using a standard protocol (Griguoli et al., 2010). Briefly, after being anesthetized with CO_2_, animals were decapitated. The brain was quickly removed from the skull and placed in ice-cold artificial cerebrospinal fluid (ACSF) containing (in mM): 130 NaCl, 25 glucose, 3.5 KCl, 1.2 NaH_2_PO_4_, 25 NaHCO_3_, 2 CaCl_2_, and 1.3 MgCl_2_, saturated with 95% O2 and 5% CO_2_ (pH 7.3–7.4). Transverse hippocampal slices were cut with a vibratome and stored at room temperature (22–24°C) in a holding bath containing the same solution as above. After incubation for at least 45 min, an individual slice was transferred to a submerged recording chamber and continuously superfused at 33–34°C with oxygenated ACSF at a rate of 3–4 ml/min.

### Electrophysiological recordings

Recordings were made with a patch-clamp amplifier (Axopatch 1D amplifier, Axon Instruments, Sunnyvale, CA, USA) from CA3 pyramidal cells visualized with an upright microscope equipped with differential interference contrast optics and infrared video camera, using the whole cell configuration of the patch-clamp technique. Patch electrodes were pulled from borosilicate glass capillaries (Hingelberg, Malsfeld, Germany); when filled with an intracellular solution they had a resistance of 4–6 MΩ. The stability of the patch was checked by repetitively monitoring the input and series resistance during the experiments. Cells exhibiting 15% changes were excluded from the analysis. The series resistance was <25 MΩ.

Spontaneous glutamatergic and GABAergic postsynaptic currents were routinely recorded from a holding potential of −65, −70 mV in the presence of bicuculline (10 μM) and DNQX (20 μM), respectively. Miniature currents were recorded in the presence of TTX (1 μM) to block sodium currents and propagated action potentials. For glutamatergic currents we used an intracellular solution containing (in mM): 125 Cs-methanesulphonate, 10 CsCl, 10 HEPES, 0.3 EGTA, 2 MgATP, 0.3 NaGTP, (pH adjusted to ~7.3 with CsOH). For GABAergic currents we used an intracellular solution containing (in mM): CsCl 137, Hepes 10, BAPTA 11, MgATP 2, MgCl_2_ 2, CaCl_2_ 1 and 5 QX-314 (pH adjusted to ~7.3 with CsOH).

Concentric bipolar electrodes were used to stimulate granule cells in the dentate gyrus in order to elicit 2-amino-3-(5-methyl-3-oxo-1,2-oxazol-4-yl) propanoic acid (AMPA)-mediated excitatory postsynaptic currents (EPSCs) in CA3 pyramidal neurons (frequency of stimulation: 0.1 Hz; stimulus duration 100–200 μs). Stimulus strength was adjusted to obtain at −65 mV stable EPSCs of ~100 pA amplitude. The NMDA component was recorded from the same neuron at +40 mV, using the same stimulus strength, after blocking the AMPA-mediated component with DNQX (20 μM). While AMPA-mediated EPSCs were recorded close to the reversal potential of GABA, NMDA currents were elicited in the presence of DNQX (20 μM) and bicuculline (10 μM) to block AMPA and GABA_A_ receptors, respectively.

In some experiments, extracellular field potentials were recorded using conventional glass microelectrodes (tip diameter 5–10 μM) filled with ACSF and placed into the stratum pyramidale of the CA3 area.

### Drugs

The following drugs were used: 6,7-Dinitroquinoxaline-2,3-dione (DNQX), 6-Imino-3-(4-methoxyphenyl)-1(6*H*)-pyridazinebutanoic acid (SR 95531) hydrobromide, picrotoxin and bicuculline, purchased from Ascent Scientific; (1,2,5,6-Tetrahydropyridin-4-yl)met hylphosphinic acid (TPMPA) and *N*-(2,6-Dimethylphenylcarbamoylmethyl) triethylammonium bromide (QX 314), purchased from Tocris Bioscence; tetrodotoxin (TTX) from Latoxan. Bumetanide and 1-[2-[[(Diphenylmethylene)imino]oxy]ethyl]-1,2,5,6-tetrahydro-3-pyridinecarboxylic acid hydrochloride hydrochloride (NO-711), from Sigma-Aldrich. Zolpidem was a gift of Dr. A. Barberis (Italian Institute of Technology, Genova). Stock solutions were made in distilled water and then aliquoted and frozen at −20°C. DNQX and picrotoxin were dissolved in dimethylsulfoxide (DMSO). The final concentration of DMSO in the bathing solution was 0.1%. At this concentration, DMSO alone did not modify the membrane potential, input resistance, or the firing properties of CA3 pyramidal neurons. Drugs were applied in the bath by gravity by changing the superfusion solution to one differing only in its content of drug(s). The ratio of flow rate to bath volume ensured complete exchange within 2–3 min.

### Data analysis

Data were acquired at 20 kHz, filtered with a cut-off frequency of 2 kHz and stored on computer in order to perform off-line analysis. Miniature AMPA and GABA_A_-mediated postsynaptic currents were analyzed using pClamp 10.0 (Molecular Devices, Sunnyvale, CA, USA). This program uses a detection algorithm based on a sliding template. The template did not induce any bias in the sampling of events because it was moved along the data trace by one point at a time and was optimally scaled to fit the data at each position. The detection criterion was calculated from the template-scaling factor and from how closely the scaled template fitted the data.

To minimize the contribution of the unquantifiable effects of cable filtering, detailed kinetic analysis of miniature synaptic currents was limited to events with a rise time ≤1 ms. The rise time was estimated as the 10–90% time needed to reach the peak of the synaptic current. The weighted decay was obtained by dividing miniatures charge transfer by their amplitude.

The amplitude of the tonic current was estimated by the outward shift of the baseline current after the application of the GABA_A_ receptor channel blocker picrotoxin (100 μM). Only current recordings that exhibited a stable baseline were included in the analysis. Baseline currents and their standard deviations were estimated by plotting 10 s periods of raw data in all point histograms. These were fitted with a Gaussian function. The peak of the fitted Gaussian was considered as the mean holding current (Glykys and Mody, 2007).

NMDA/AMPA ratio was measured by averaging 15 to 30 sweeps for each holding potential. The AMPA-mediated component was measured at the peak of the current obtained at −65 mV, while the NMDA component was measured at the peak of the current obtained at +40 mV after blocking the AMPA component with DNQX. The weighted decay time constant for NMDA-mediated synaptic current was measured by dividing the area by its amplitude, independently of the fitting (Cathala et al., 2003).

Peak scaled non-stationary noise analysis was performed according to Traynelis et al. (1993) using the Mini Analysis program (version 6.0.1, Synaptosoft, Leonia, NJ). Miniature events were selected following the procedure described by Momiyama et al. (2003). Briefly, individual events were analyzed by measuring the peak amplitude, the 10–90% rise time and the decay time constant. Measured parameters were then numbered according to the event number and tested by Spearman's rank order correlation test for time stability. After testing for correlation between rise time and amplitude and between rise time and decay time, miniature GABAergic events were used for noise analysis. Individual events were aligned to the point of steepest rise time. The peak of the mean current response waveform was scaled to the response value at the corresponding point in time of each individual event before subtraction to generate the difference waveforms. The ensemble mean post synaptic current was binned into 50 bins of equal amplitude to assign similar weights to all phases of ensemble mean waveform. Variance was plotted against amplitude and individual points were fitted with the equation:

(1) $$\sigma^{2}(I)=iI-I^{2}/N+\sigma_{b}^{2}$$

where *i* is the unitary single-channel current, *I* is the mean current, *N* is the number of channels open at the current peak and σ^2^_b_ is the variance of the background noise. The single-channel chord conductance (γ) was calculated as:

(2) $$\gamma =i/\text{​}\left(E_{m}-E_{\text{rev}}\right)$$

from the holding potential (*E*_*m*_) of −70 mV, assuming a reversal potential (*E*_*rev*_) of 0 mV.

### Statistical analysis

All values are presented as mean ± SEM. In most of the experiments, statistical comparison was performed using the unpaired *t*-test. Due to the non-Gaussian distribution in the frequency and amplitude of miniature GABAergic and glutamatergic events, probability plots were compared using the Kolmogorov–Smirnov test. Significance between independent experimental groups was calculated using the One-Way ANOVA. Bonferroni post test was used to evaluate the statistically significance between paired groups within the multiple comparison. A *p*-value <0.05 was considered as statistically significant.

## Results

### Spontaneous network-driven events are enhanced in NL3^R451C^ mice

We first examined the resting membrane potential (*V*_rest_) and the input resistance (*R*_in_) of the recording neurons (at P4–P9). No significant differences in these parameters were found between WT and NL3^R451C^ mice (*V*_rest_: −50 ± 2 mV and −44 ± 3 mV in WT and NL3^R451C^ mice, respectively; *R*_in_: 815 ± 200 MΩ and 770 ± 162 MΩ in WT and NL3^R451C^ mice, respectively; *n* = 13; *p* > 0.05 for both *V*_rest_ and *R*_in_).

As ASDs are neurodevelopmental disorders, we first verified whether the NL3^R451C^ mutation alters correlated network activity, the so-called Giant Depolarizing Potentials (GDPs), a hallmark of developmental networks (Ben-Ari et al., 1989, 2007). GDPs which represent a primordial form of synchrony between neurons, thought to be essential for proper circuit maturation (Ben-Ari et al., 2007; Blankenship and Feller, 2010), are generated by the synergistic action of glutamate and GABA, both of which are depolarizing and excitatory at early development stages (Cherubini et al., 1991).

**Figure 1:**
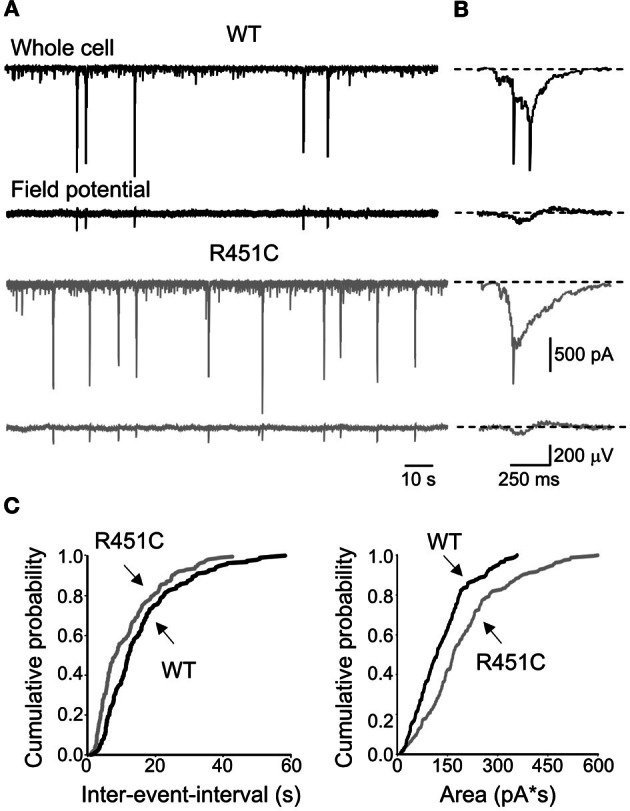
**The NL3^R451C^ knock-in mutation enhances correlated network activity in the immature hippocampus. (A)** Whole cell voltage-clamp (upper traces) and extracellular field recordings (lower traces) of GDPs recorded at P5 in hippocampal slices obtained from WT (black) and NL3^R451C^ mice (gray). **(B)** GDPs from the traces in **(A)** shown on an expanded time scale. **(C)** Cumulative probability plots of inter-event intervals and areas of inward currents underlying GDPs recorded from WT (*n* = 24) and NL3^R451C^ mice (*n* = 17) between P4 and P9. Curves in the plots referring to WT and R451C KI mice are significantly different (*p* < 0.01; *p* < 0.01; K–S test).

We focused on the CA3 region of the hippocampus where GABAergic interneurons are centrally involved in GDPs generation (Bonifazi et al., 2009; Picardo et al., 2011; Allene et al., 2012). GDPs were recorded at P4–P9 simultaneously from single cells and from population of neurons as extracellular field potentials (Figures 1A,B).

GDPs occurred more frequently in NL3^R451C^ knock-in mice respect to WT (0.69 ± 0.009 Hz, *n* = 17, and 0.046 ± 0.006 Hz, *n* = 24 in knock-in and control mice, respectively; *p* < 0.05; unpaired *t*-test). As shown in the cumulative inter-event-interval plot of Figure 1C, the curve obtained from NL3^R451C^ knock-in mice was shifted to the left respect to that obtained from WT animals (*p* < 0.05; K–S test). In addition, as illustrated in Figure 1C, in NL3^R451C^ knock-in mice the cumulative probability curve related to charge transfers through the currents underlying GDPs was shifted to the right respect to controls and was significantly different from that obtained from WT animals (*p* < 0.01; K–S test). In accord with a previous study (Le Magueresse et al., 2006), GDPs became rare at P8–9 and disappeared after P10 in both WT and NL3^R451C^ knock-in mice. To assess whether the depolarizing action of GABA was instrumental in triggering GDPs in both WT and NL3^R451C^ knock-in mice, we tested the consequences of converting GABA action from depolarizing to hyperpolarizing by exposing the slices to bumetanide, a selective blocker of the cation–chloride cotransporter NKCC1, responsible for the accumulation of chloride inside the cell (Dzhala et al., 2005; Sipila et al., 2006; Safiulina et al., 2008). In agreement with a previous study (Safiulina et al., 2008), bumetanide (10 μM) completely abolished GDPs recorded at P4–P9 from WT (*n* = 5) and NL3^R451C^ knock-in (*n* = 3) mice (data not shown), indicating that during the first postnatal days the depolarizing action of GABA contributes to generate GDPs in both strains of animals.

### Enhanced basal gabaergic but not glutamatergic transmission in young NL3^R451C^ knock-in mice

**Figure 2:**
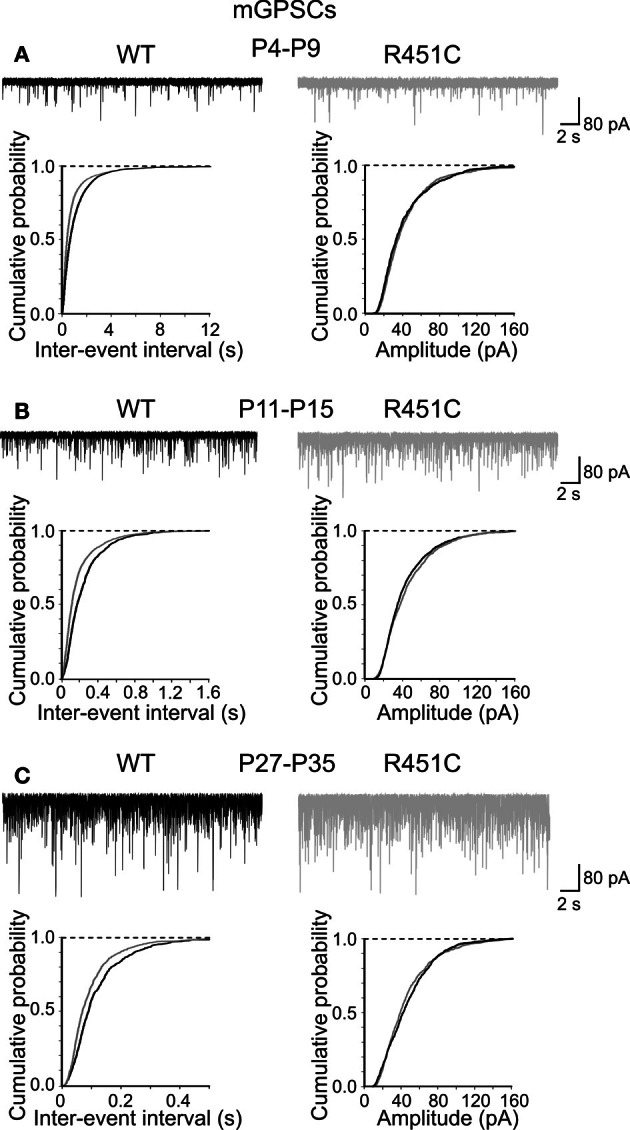
**The NL3^R451C^ knock-in mutation enhances the frequency of mGPSCs**. Samples traces of mGPSCs recorded from CA3 principal cells at P4–P9 **(A)**, P11–P15 **(B)** and P27–P35 **(C)** from WT (black) and in NL3^R451C^ knock-in mice (gray). Below, cumulative probability plots of inter-event intervals (left) and amplitude (right) of obtained from WT and NL3^R451C^ knock-in mice. Changes in inter-event-interval (*p* < 0.05; K–S test) but not in amplitude (*p* > 0.05; K–S test) were significantly different at all developmental stages examined.

Since network dynamics rely on neuronal connectivity and GDPs are strictly dependent on the synergistic action of GABA and glutamate, both depolarizing and excitatory (Ben-Ari et al., 2007), we next examined whether changes in spontaneous miniature synaptic currents could account for the observed effects. Miniature GABA_A_- and AMPA-mediated postsynaptic currents (mGPSCs and mEPSCs, respectively) were recorded at three different postnatal ages (P4–P9, P11–P15, P27–P35) in the presence of TTX (1 μM) and DNQX or bicuculline, respectively. As shown in Figure 2, significant differences in the inter-event intervals distributions (but not amplitude) of mGPSCs were observed in all groups examined (*p* < 0.05 for all three groups; K–S test).

**Figure 3:**
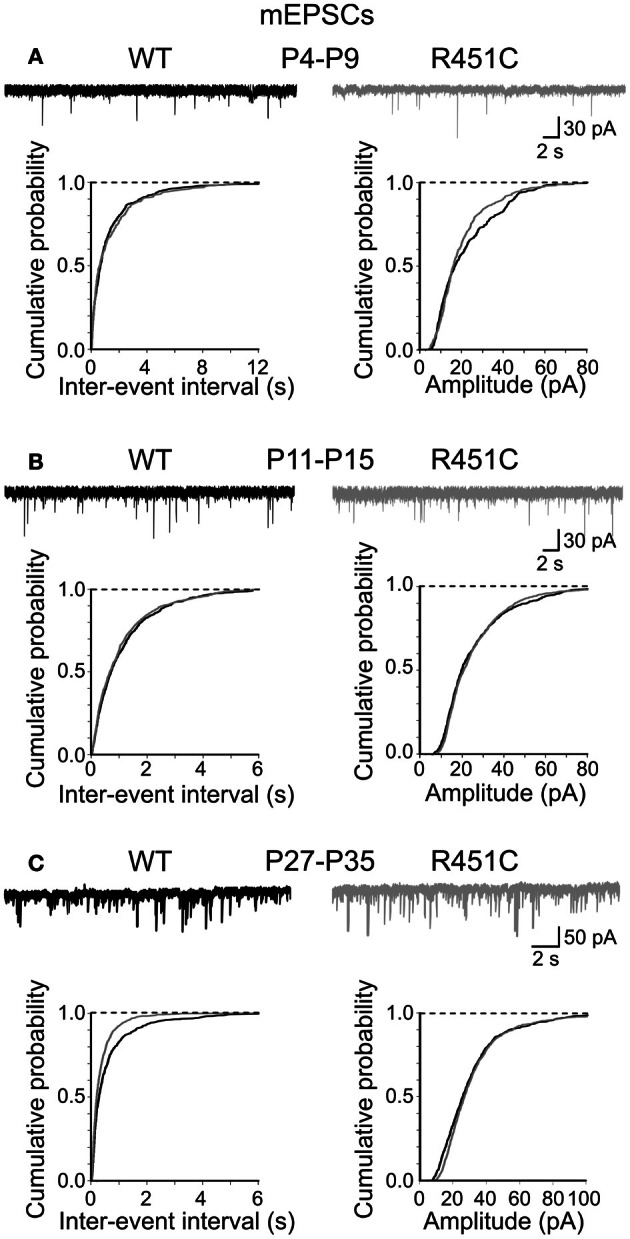
**The NL3^R451C^ knock-in mutation does not affect spontaneous miniature glutamatergic events. (A)** Samples traces of mEPSCs recorded from CA3 principal cells at P4–P9 from WT (black) and in NL3^R451C^ knock-in mice (gray). Below, cumulative probability plots of inter-event intervals (left) and amplitude (right) of obtained from P4–P9 **(A)**, P11–P15 **(B)**, and P27–P35 **(C)** WT and NL3^R451C^ knock-in mice. **(B)** and **(C)** as **(A)** but for later developmental stages. Note changes in frequency (but not amplitude of miniature events at P27–P35 (the two curves are significantly different; *p* < 0.05; K–S test).

In contrast, cumulative distribution plots of inter-event-intervals of mEPSCs recorded from NL3^R451C^ and WT mice were significantly different only at P28–P35 (*p* < 0.05; K–S test; Figure 3). No significant differences in cumulative amplitude distributions were observed between NL3^R451C^ and WT mice (*p* > 0.05; K–S test).

Altogether, these results strongly support the involvement of the NL3^R451C^ mutation in the enhancement of GABAergic transmission at early stages of postnatal development.

Furthermore, to assess whether the NL3^R451C^ mutation differentially affects AMPA- and NMDA-mediated synaptic transmission, AMPA- and NMDA-mediated postsynaptic currents evoked by granule cell stimulation were examined at P4–P9 from CA3 principal cells at −65 mV and +40 mV, respectively. While AMPA-mediated EPSCs were recorded close to the reversal potential of GABA, NMDA currents were elicited in the presence of DNQX (20 μM) and bicuculline (10 μM) to block AMPA and GABA_A_ receptors, respectively. No significant differences were observed in the NMDA/AMPA ratio between the two different genotypes (WT 0.24 ± 0.06, *n* = 6; KI 0.28 ± 0.04 *n* = 4; *p* > 0.05; data not shown).

In addition, similar decay time values of NMDA-mediated EPSCs were detected in both genotypes (WT 88.17 ± 6 ms, *n* = 6; NL3^R451C^ 85.1 ± 20 ms; *n* = 4; *p* > 0.05), indicating that at MF-CA3 synapses the NL3^R451C^ mutation does not alter glutamatergic synaptic transmission and the postsynaptic expression of NMDA receptor subunits.

In the following experiments we focused on the second postnatal week (P11–P15) which corresponds to the period of maximal synaptogenesis and NLs expression (Budreck and Scheiffele, 2007).

### The NL3^R451C^ knock-in mutation affects GABA release

According to the quantal theory, the synaptic efficacy *E*, the mean amplitude of unitary GPSCs, can be defined as *E* = mQ, where *m* is the quantal content or the mean number of quanta released per presynaptic action potential and *Q* is the quantal size or amplitude of the unitary postsynaptic current (Katz, 1969). While *Q* depends on both pre (GABA content in synaptic vesicles) and postsynaptic (GABA_A_ receptors) mechanisms, *m* depends on presynaptic factors, namely the number of release sites *N* and the probability of release (*P*) at each individual site. Therefore, presynaptic changes in GABA release may be related to modifications either in *Q* or *m* or both.

**Figure 4:**
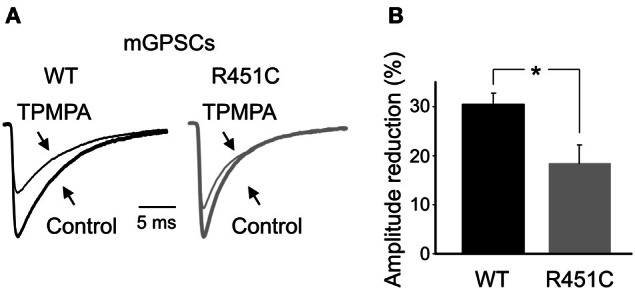
**Increased synaptic GABA transient in NL3^R451C^ knock-in mutant mice. (A)** Sample traces of mGPSCs recorded from NL3^R451C^ mutants (gray) and littermate (black), in the absence (tick line, Control) and in the presence of TPMPA (200 μM; thin line). The amplitudes of mGPSCs from NL3^R451C^ knock-in mice were normalized to those obtained from WT animals. **(B)** Each column represents the mean TPMPA-induced reduction in amplitude of mGPSCs in WT (black; *n* = 8) and NL3^R451C^ mice (gray; *n* = 7). ^*^ *p* < 0.05.

To assess whether the increase in frequency of mGPSCs observed in NL3^R451C^ mice is due to changes in GABA release, we analyzed GABA transients in the cleft. We took advantage of the low affinity competitive GABA_A_ receptor antagonist TPMPA which has a very fast dissociation constant and competes with synaptically released GABA for the same ligand binding site on GABA_A_ receptors (Jones et al., 2001; Barberis et al., 2004). This allowed us to compare differences in presynaptic GABA release between WT and NL3^R451C^ mice, because the sensitivity of mGPSCs to TPMPA block is strongly influenced by relative changes in GABA concentration, as both GABA and TPMPA compete for the same binding sites (Mozrzymas et al., 1999; Barberis et al., 2000; Jones et al., 2001). Bath application of TPMPA (200 μM) caused a more pronounced reduction of mGPSCs amplitude in WT than in NL3^R451C^ mice (30.5 ± 2.2%; *n* = 8 and 18.4 ± 3.7%; *n* = 7 in WT and NL3^R451C^ mice, respectively, *p* < 0.05; One-Way ANOVA and Bonferroni post test for multi comparison analysis between groups), suggesting an increased GABA transient in the synaptic cleft of NL3^R451C^ mice respect to controls (Figure 4).

To test whether this effects was associated with an increased number of available GABA_A_ receptors on the postsynaptic membrane (opened by a single GABA quantum, on the assumption that GABA_A_ receptors are not saturated by the content of a single vesicle, Barberis et al., 2004), we used the peak-scaled non-stationary fluctuation analysis (see Methods; Traynelis et al., 1993). We used only stable recordings of mGPSCs with no time-dependent changes in either peak amplitude, 10–90% rise time and decay time (electrotonic filtering was excluded on the basis of no correlation between the 10–90% rise time and the decay time; Momiyama et al., 2003).

**Figure 5:**
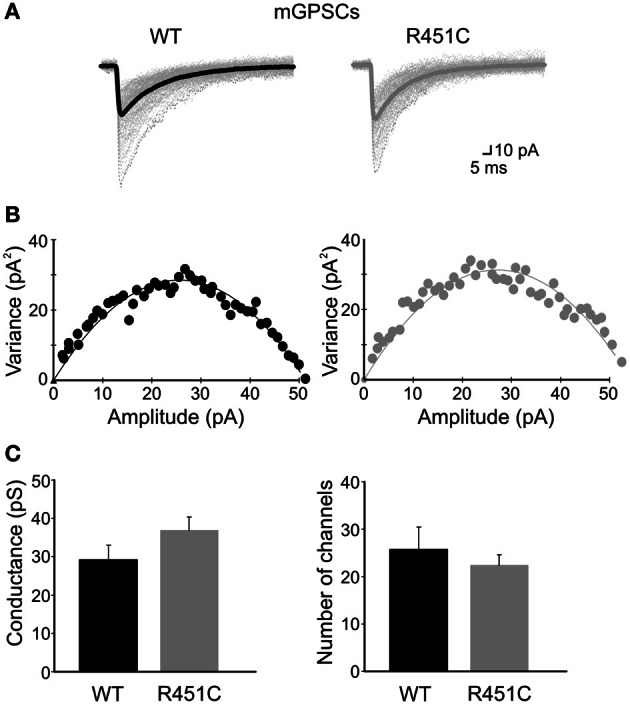
**The NL3^R451C^ knock-in mutation does not affect single channel conductance and/or the number of GABA_A_ receptor channels. (A)** Individual mGPSCs (dotted lines) are shown with the average currents (thick lines) in WT and R451C mice. **(B)** Current/variance relationships for mGPSCs shown in **(A)**. **(C)** Summary plots of weighted mean channel conductance and number of GABA_A_ receptor channels in WT (black; *n* = 8) and in NL3^R451C^ knock-in mice (gray; *n* = 9).

For each cell a parabolic variance vs. mean curve was obtained (see individual samples in Figures 5A,B). By fitting data points with Equation 1 gave an estimated unitary currents of 2 ± 0.27 pA and 2.6 ± 0.25 pA in WT and NL3^R451C^ mice, respectively, corresponding to a weighted mean channel conductance of 29.2 ± 3.8 pS (*n* = 8) and 37 ± 3.5 pS (*n* = 9). These values were not significantly different (*p* > 0.05; Figure 5C). In addition, no significant differences in the number of GABA_A_ receptor channels were found between WT and knock-in mice (25.7 ± 4.8, *n* = 8 and 22.4 ± 2.1, *n* = 9, in WT and NL3^R451C^ mice, respectively; *p*>0.05; Figure 5C) indicating that changes in frequency of mGPSCs could not be attributed to modifications in the number of receptor channels opened at the peak of a spontaneous miniature GABAergic events.

### The NL3^R451C^ knock-in mutation alters the decay kinetic of GABA_A_ receptors

**Figure 6:**
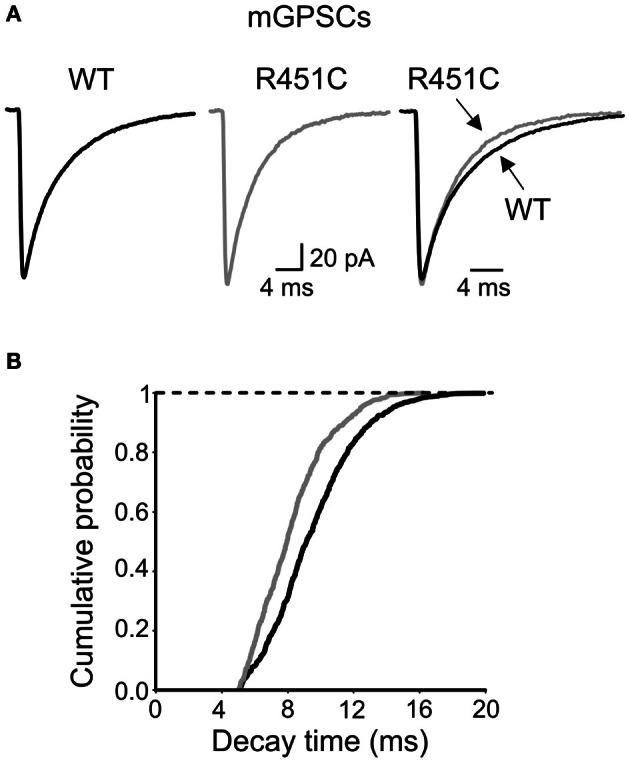
**Faster decay of mGPSCs in NL3^R451C^ knock-inmice. (A)** Sample traces of mGPSCs (each trace is the average of >50 individual traces) obtained from NL3^R451C^ knock-inmice (gray) and control littermates (WT, black). On the right the two traces are superposed. **(B)** Cumulative probability plot of decay times from WT (black; *n* = 9) and NL3^R451C^ knock-inmice (gray; *n* = 8). The two curves are significantly different (*p* < 0.01; K–S test).

Next, we examined the kinetic properties of miniature GABAeric events. As shown in Figure 6A, respect to WT animals, synaptic currents obtained from NL3^R451C^ KI mice at P9–P11 displayed a significantly (*p* < 0.05) faster decay time (the weighted decay time was 9.14 ± 0.4 ms; *n* = 9; and 7.43 ± 0.5 ms; *n* = 9; in WT and NL3^R451C^ mice, respectively; in the absence of any change in the rise time (0.53 ± 0.02 ms; *n* = 9; and 0.47 ± 0.02 ms; *n* = 9; in WT and NL3^R451C^ mice, respectively; *p* > 0.05). This led to a leftward shift of the cumulative probability curve obtained from NL3^R451C^ respect to WT mice (the two cumulative curves were significantly different; *p* < 0.05, K–S test; Figure 6B).

The faster decay time of mGPSCs observed in NL3^R451C^ mice may result from a differential expression of GABA_A_ receptor subunits in the postsynaptic membrane. One possibility is that the NL3^R451C^ KI mutation accelerates the developmental switch from α2 to α1 subunits of GABA_A_ receptors, known to produce currents with faster decay kinetics (Laurie et al., 1992; Cherubini and Conti, 2001). To elucidate whether in NL3^R451C^ mice more α1 containing GABA_A_ receptors are recruited at synapses respect to α2, we examined the prolongation of current decay induced by zolpidem, known to selectively enhance the activity of α1 subunit-containing receptors (Pritchett and Seeburg, 1990). Since the effects of zolpidem on miniature GABAergic currents reflects the degree of receptor occupancy (Perrais and Ropert, 1999), we analyzed only mGPSCs duration. Application of zolpidem (100 nM) prolonged the decay time of mGPSCs in both WT and NL3^R451C^ mice. Although zolpidem-induced prolongation of synaptic decay was more pronounced in NL3^R451C^ KI micerespect to controls(NL3^R451C^, 23 ± 4%, *n* = 7 WT, 14 ± 2%, *n* = 6; data not shown) this effect did not reach a significant level (*p* = 0.2, One-Way ANOVA and Bonferroni post test for multi comparison analysis between groups).

### The NL3^R451C^ KI mutation does not alter the tonic GABA_A_-mediated conductance

Once released, GABA rapidly diffuses across the synaptic cleft to occupy synaptic GABA_A_ receptors. Part of the neurotransmitter escapes the cleft and invades the extracellular space to occupy extrasynaptic high affinity receptors and to generate a persistent GABA_A_-mediated conductance (Farrant and Nusser, 2005) which is involved in a number of physiological and pathological processes (Brickley and Mody, 2012). Recent studies have demonstrated a down-regulation of GABA_A_-mediated tonic conductance in an animal model of X Fragile syndrome, a common inherited cause of mental retardation with language deficit and autistic behavior (Curia et al., 2009; Olmos-Serrano et al., 2010). Therefore, in the following experiments we searched for differences in GABA_A_-mediated tonic conductance between WT and NL3^R451C^ mice. The tonic conductance was obtained by measuring the shift in the holding current following the application of the GABA_A_ receptor channel blocker picrotoxin (100 μM). This drug caused a similar shift in holding current in WT and in NL3^R451C^ mice (54 ± 11 pA, *n* = 11, and 49 ± 15 pA, *n* = 6, in WT and in NL3^R451C^ mice, respectively; *p* = 0.8; data not shown).

**Figure 7:**
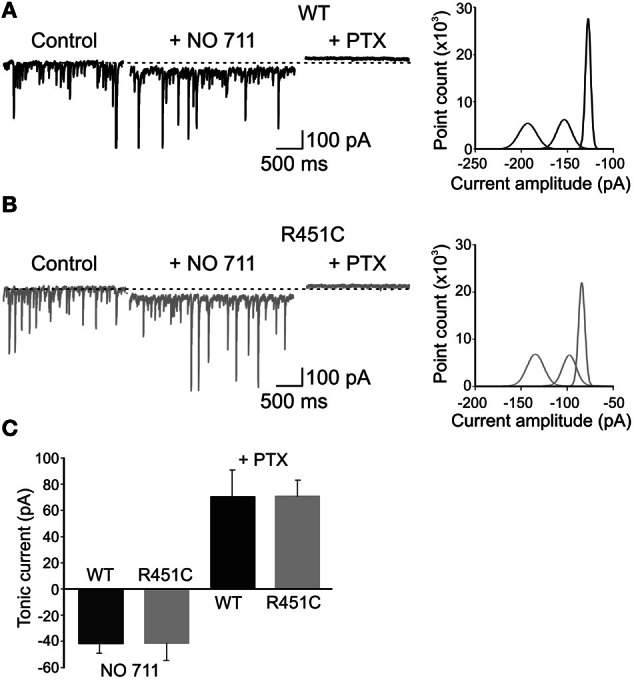
**The NL3^R451C^ knock-in mutation does not alter GABA transporters and tonic GABA_A_-mediated inhibition. (A)** Left: representative traces of spontaneous GPSCs recorded in a P 12 pyramidal cell in hippocampal slice obtained from a WT animal before (Control), during application of the GAT-1 blocker NO-711 (10 μM) and NO-711 plus picrotoxin (100 μM). Right: all-point histogram of 10 ms traces from the cell recorded on the left in control conditions, in the presence of NO-711 and NO-711 plus picrotoxin. **(B)** As in **(A)** but from NL3^R451C^ KI mutant mouse. **(C)** Summary data obtained from WT (black; *n* = 6) and NL3^R451C^ knock-in mutant mice (gray; *n* = 7).

Furthermore, to test whether GABA transporters differentially affect ambient GABA in WT and NL3^R451C^ mice, we applied NO-711, which selectively blocks the neuronal and glial GABA transporter GAT-1 (Borden, 1996; Semyanov et al., 2004). NO-711 (10 μM) produced a similar inward shift of the baseline current in both WT and NL3^R451C^ mice (41.5 ± 12.8 pA and 42 ± 7.5 pA in WT, *n* = 6, and knock-in animals, respectively; *n* = 7; *p* = 0.7 in both; Figures 7A–C). Addition of picrotoxin caused in the two genotypes a similar outward shift in baseline currents (71 ± 20 pA; *n* = 6 and 71 ± 12 pA; *n* = 7; in WT and in NL3^R451C^ mice, respectively, *p* = 0.98). These data indicate that the NL3^R451C^ mutation does not affect GABA transporters and the tonic GABA_A_-mediated conductance.

## Discussion

The present results provide evidence that the NL3^R451C^ mutation selectively affects correlated network activity and GABAergic signaling in the hippocampus already at birth. A previous study from layer 2/3 pyramidal neurons in acute slices of somatosensory cortex obtained from juvenile (P13–P16) NL3^R451C^ KI mice has revealed an increased inhibitory synaptic transmission. These animals exhibited enhanced spatial learning abilities associated with deficits in social interaction [(Tabuchi et al., 2007); but see Chadman et al. (2008) for the behavioral phenotype], reminiscent of those found in some patients affected by ASD. The authors suggested that the NL3 mutation enhances GABAergic transmission without changing the release probability since they failed, at least in the barrel cortex, to detect major modifications in short-term synaptic plasticity.

Our data on GDPs indicate that the NL3^R451C^ mutation affects GABA release. During the first week of postnatal life, GDPs are generated by the interplay between GABA and glutamate, both of them depolarizing and excitatory. Therefore, changes in frequency and shape of spontaneous giant events can be attributed to modifications of the GABAergic, glutamatergic drive to principal cells or in both. A close examination of spontaneous miniature events, occurring during the first week of postnatal life, revealed an increase in frequency, but not in amplitude of mGPSCs, suggesting a presynaptic type of action. This was further supported by TPMPA experiments that, as expected for an enhanced GABA transient in the cleft, showed a reduced blocking effect of the fast-off GABA_A_ antagonist on miniature events in NL3^R451C^ KI mice.

Although an increased number of available postsynaptic GABA_A_ receptors, may account for these results (on the assumption that these are not saturated by the release of GABA from a single vesicle; Barberis et al., 2004; Hartman et al., 2006), this was not the case since a similar number of receptor channels was revealed with peak-scaled non-stationary fluctuation analysis in both WT and NL3^R451C^ knock-in mice. Presynaptic changes in GABA release can be attributed to modifications in the probability of GABA release or in the number of release sites. Considering that miniature events are generated by the release of a single quantum, it seems more likely that an increased number of release sites contributes to the observed effects. This is supported by previous data from Südhof group showing an enhancement of the presynaptic GABAergic marker VGAT (but not VGlut1) in the hippocampus of NL3^R451C^ KI mice (Tabuchi et al., 2007) respect to controls.

In the present experiments we did not characterize which subtype of GABAergic interneuron was involved in the observed effects. Although parvalbumin-positive basket cells certainly contribute to the spontaneous action potential-independent release of GABA (Freund and Katona, 2007) we cannot exclude the participation of other interneuron subtypes. In the hippocampus, parvalbumin-positive cells, already present at birth (Bonifazi et al., 2009) play a crucial role in coordinating the timing of neuronal activity, thus contributing to generate theta and gamma rhythms involved in high cognitive functions (Bartos et al., 2007; Klausberger and Somogyi, 2008; Wulff et al., 2009). In some animal models of ASDs (NL3^R451C^ knock-in mice and rats prenatally exposed to the histone deacetylase inhibitor, valproate) an asymmetric reduced expression of parvalbumin-positive interneurons across hemispheres has been detected (Gogolla et al., 2009). Since parvalbumin-positive neurons normally drive experience-dependent circuit development (Fagiolini et al., 2004; Hensch, 2005), the selective disruption of these cells may alter neuronal networks during a critical period of postnatal development (Pizzarelli and Cherubini, 2011). Whatever is the subtype of GABAergic interneuron involved, the present data unveil an alteration of the excitatory/inhibitory balance, known to exert a key role in the refinement of cortical circuits early in postnatal life (LeBlanc and Fagiolini, 2011).

It is worth noting that at P27–P35 (and not at early developmental stages), the increased frequency of miniature GABAergic currents paralleled that of miniature glutamatergic events. Although the cause of this effect is presently unclear, as a matter of speculation we can hypothesize that this represents a form of compensatory mechanism developed to counter the early impairment of GABAergic transmission. In this respect, the possibility that changes in the expression of AMPA- and NMDA-receptors containing the NR2B subunits, observed in the adult hippocampus may underlie the same phenomenon, cannot be excluded (Etherton et al., 2011). Alternatively, we cannot exclude that the NL3 mutation affects glutamatergic synaptic transmission only at late developmental stages when reactive plasticity is more pronounced (Groc et al., 2006).

Interestingly, as compared to WT animals, NL3^R451C^ knock-in mice exhibited mGPSCs with faster decay kinetics, suggesting a possible postsynaptic effect (Barberis et al., 2011). One possibility is that the NL3 mutation affects receptor trafficking, facilitating the recruitment of α1 receptor containing subunits at mutated synapses. However, the lack of significantly different responses to zolpidem between WT and NL3^R451C^ KI mice allows excluding this possibility. Although other receptor subtypes may account for the observed effects, we cannot exclude that a fast entrance into the desensitized state of GABA_A_ receptors exposed to an increased amount of GABA may account for the acceleration of mGPSCs decay in NL3^R451C^ KI mice.

How does the E/I balance regulate brain functions? Recent studies have highlighted the role of trans-synaptic signaling *via* NLs and Nrxs in assembling and stabilizing pre and postsynaptic components (Südhof, 2008). In particular, the NLs which in vertebrate are encoded by four genes (*Nlgn1–4* with various splice variants) form homo-dimers through their extracellular domains (Missler et al., 2012). While NL1 is preferentially associated with glutamatergic synapses (Song et al., 1999), NL2 and NL4 with GABAergic synapses (Graf et al., 2004; Varoqueaux et al., 2004; Dong et al., 2007; Hoon et al., 2011). Interestingly, we have recently found that gephyrin, a core protein of inhibitory postsynaptic densities that interacts with the cytoskeleton to stabilize inhibitory receptors in precise opposition to presynaptic active zones, transynaptically acts *via* NL2 on GABA release, thus directly contributing to maintain an appropriate E/I balance (Marchionni et al., 2009; Varley et al., 2011). Hampering gephyrin function not only alters GABA_A_ receptors clusterization and their gating properties but also the probability of GABA release, an effect mediated by NL2 since it could be rescued by over expressing this protein in gephyrin-deprived neurons (Varley et al., 2011). Although NL3 is highly expressed in the brain where is localized at both excitatory and inhibitory synapses (Budreck and Scheiffele, 2007), its functional role remains to be elucidated. However, the developmental pattern of NL3 expression, whose peak is coincident with that of synaptogenesis strongly suggests the involvement of this protein in synapse formation and stabilization.

It is worth noting that although mutated NL genes or associated proteins have been found only in a small number of young patients, they provide crucial information on the synaptic abnormalities which possibly affect ASDs.

## Author contributions

Enrico Cherubini and Rocco Pizzarelli: conceived and designed the experiments. Rocco Pizzarelli: performed the experiments, analyzed data. Enrico Cherubini: wrote the paper. Both authors approved the final version of the manuscript.

### Conflict of interest statement

The authors declare that the research was conducted in the absence of any commercial or financial relationships that could be construed as a potential conflict of interest.
